# Machine learning from fetal flow waveforms to predict adverse perinatal outcomes: a study protocol

**DOI:** 10.12688/gatesopenres.12796.1

**Published:** 2018-02-12

**Authors:** Zahra Hoodbhoy, Babar Hasan, Fyezah Jehan, Bart Bijnens, Devyani Chowdhury

**Affiliations:** 1 Department of Paediatrics and Child Health, Aga khan University, Karachi, 75500, Pakistan; 2 Department of Information and Communication Technologies, Pompeu Fabra University, Barcelona , Catalonia , 08002, Spain; 3 ICREA, Barcelona, Spain; 4 Cardiology Care for Children, Lancaster, PA, 17601, USA

**Keywords:** echocardiography, pregnancy, machine learning, adverse outcomes

## Abstract

**Background:** In Pakistan, stillbirth rates and early neonatal mortality rates are amongst the highest in the world. The aim of this study is to provide proof of concept for using a computational model of fetal haemodynamics, combined with machine learning. This model will be based on Doppler patterns of the fetal cardiovascular, cerebral and placental flows with the goal to identify those fetuses at increased risk of adverse perinatal outcomes such as stillbirth, perinatal mortality and other neonatal morbidities.

**Methods:** This will be prospective one group cohort study which will be conducted in Ibrahim Hyderi, a peri-urban settlement in south east of Karachi. The eligibility criteria include pregnant women between 22-34 weeks who reside in the study area. Once enrolled, in addition to the performing fetal ultrasound to obtain Dopplers, data on socio-demographic, maternal anthropometry, haemoglobin and cardiotocography will be obtained on the pregnant women.

**Discussion:** The machine learning approach for predicting adverse perinatal outcomes obtained from the current study will be validated in a larger population at the next stage. The data will allow for early interventions to improve perinatal outcomes.

## Introduction

A pregnant woman and her newborn are most vulnerable around the time of delivery and the postnatal period ^1^. Stillbirths and early neonatal deaths within 7 days of life are a major cause of perinatal mortality. In 2013, 2.8 million neonatal deaths ^2^ and an equal number of stillbirths occurred globally ^3^. The majority of all stillbirths and neonatal deaths occur in low-income and middle-income countries in south Asia and sub-Saharan Africa ^3^. Pakistan is one of the countries where the stillbirth rate (43/1000 total births) and neonatal mortality rate (55/1000 live births) are among the highest in the world ^4^. The figures for perinatal mortality remained unchanged between Pakistan Demographic and Health Survey (PDHS) 1990–91 and PDHS 2012–13 ^4^. These numbers highlight the importance of evaluating new techniques to identify high-risk pregnant women who can then subsequently receive personalized care to improve perinatal outcomes.

The ductus venosus, ductus arteriosus and foramen ovale, are essential shunts in the fetal circulation which play an important role in development of the fetal heart and circulation during the second and third trimester ^5^. The ductus arteriosus is a wide vessel connecting the pulmonary arterial trunk to the descending aorta with approximately 40% of the cardiac output directed through it ^6^. The ductus venosus connects the umbilical vein to the inferior vena cava at its inlet to the heart ^5^. This vessel plays a critical role in the fetal circulation because it shunts oxygenated and nutrient-rich venous blood to the brain and myocardium instead of the fetal liver ^7^. The Doppler examination of the ductus venosus is used to identify hypoxaemia, cardiac decompensation and placental compromise ^8^ with abnormal waveforms (i.e. absent or reversal of end diastolic flow) being strongly associated with perinatal mortality in fetuses with early-onset IUGR ^9^. The aortic isthmus (AoI) forms a watershed between the upper body (including the brain) and lower body (including the placenta) ^5^. Fetuses with retrograde AoI blood flow fail to shift their right ventricular output from the pulmonary to the systemic circulation, indicating cerebral hypoxia ^10^. However, there is limited knowledge regarding the pattern of redistribution in the fetal cardiovascular systems which lead to these changes ^11^.

Fetal blood flow waveforms assessed via Doppler ultrasound to indicate wellbeing of the fetus have been in practice for nearly four decades ^12^. Abnormal blood flow patterns in the fetal circulation detected by Doppler ultrasound may indicate poor fetal prognosis ^13^. A recent Cochrane review on 19 trials involving approximately 10,000 women reported fewer perinatal deaths (RR-0.71, 95% confidence interval (CI) 0.52 to 0.98), fewer inductions of labour (RR 0.89, 95% CI 0.80 to 0.99) and fewer caesarean sections (RR 0.90, 95% CI 0.84 to 0.97) in women who received Doppler ultrasound to assess fetal well-being ^13^. A recent review of approximately 55,000 women reported that flow velocity waveforms across the uterine artery have a very high specificity (93.3%; 95% CI 90.4-95.5) for identifying growth restricted fetuses ^14^.

Flow changes in several other major arteries of the feto-placental circulation, as detected by Doppler echocardiography, may provide important information regarding fetal compromise which may lead to perinatal morbidity and mortality ^11^. Doppler parameters such as umbilical artery, middle cerebral artery (MCA), aortic isthmus and ductus venosus have been used for identification, risk stratification and further management of high risk pregnancies ^15^. Fetal hypoxia and acidosis may lead to absence, or even reversal, of end diastolic flow in the umbilical artery, which is associated with high perinatal morbidity and mortality ^16^. An increase in end-diastolic flow in the MCA, considered to be a brain sparing physiological phenomenon, has been seen in pregnancies suspected of intrauterine growth restriction ^17^.

Lumped computational models have been proposed to recreate and increase our understanding regarding the regulation of the utero-placental and fetal circulation ^5^. The main advantage of these models is that changes in individual parameters (which may not be possible through *in vivo* or animal based experiments) and their effect on Doppler indices can be evaluated ^6, 11^. Machine learning models can be used to predict IUGR in the second trimester ^7, 11^. This model showed that IUGR fetuses had aortic isthmus flow changes that depend on cerebral vasodilation and increase in peripheral resistance with the latter being a major determinant, while MCA flow was mainly affected by changes in cerebral resistance ^11^. The results also suggested that patient specific haemodynamic parameters estimated with the computational model had the advantage of studying the effects of individual flow changes across the fetus and placenta which cannot be assessed in a clinical setting ^7^. This would help in understanding the pathophysiology of disease states in a more comprehensive manner and suggest possible interventions.

The Doppler flow allows us to assess the fetus that is at risk irrespective of the gestational age. It will also allow us to identify at-risk fetus without relying on anthropometric measures. As long as we are able to able to obtain the Doppler patterns on the fetus we will be able to determine if the patterns are normal or abnormal, with less of a concern of determining an accurate gestational age. In addition to obtaining fetal Dopplers, one echocardiogram will be performed on the mother. The echocardiogram will look at the four chamber view of the heart in the mother. This data may help us identify maternal etiology for poor perinatal outcomes and thus provides insights into intervention.

Though UmbiFlow (jointly developed by Medical Research Council (MRC) Unit for Perinatal Mortality, the MRC and the Centre for Scientific and Industrial Research (CSIR) South Africa), a portable device used to detect IUGR by assessing continuous wave Doppler blood flow across the placental end of the umbilical artery reported reduced number of unnecessary referrals to a higher level health facility in cases suspected of IUGR ^8^, it studies waveform across one arterial bed while our model takes into account 6 arterial beds which play a critical role in the fetal circulation.

The aim of this study is to obtain proof of concept for using a computational model of fetal hemodynamics, combined with machine learning based on Doppler patterns of the fetal cardiovascular, cerebral and placental flows, to identify those at increased risk of adverse perinatal outcomes such as stillbirth, perinatal mortality, and other neonatal morbidities. We will also compare the sensitivity and specificity of UmbiFlow with the machine learning model in predicting adverse perinatal outcomes.

## Study protocol

Study design, duration and setting – This will be a prospective one group cohort study for a period of twelve months which will be based in Ibrahim Hyderi, a peri-urban settlement of approximately 70,000 on the south east of Karachi. It is one of the seven union councils in Bin Qasim town which has a population of one million. A primary health care clinic (PHC) run by the Department of Peadiatrics and Child Health, The Aga Khan University (AKU) provides free care of early childhood illnesses and pregnancy services including antenatal checkups and free ultrasound. Pregnant women self-refer for basic ultrasound and antenatal follow-ups.

Study subjects and eligibility criteria- Pregnant women between 22–34 weeks who reside in Ibrahim Hyderi Goth. This site was chosen for their static population and the presence of demographic surveillance system setup by the Department of Pediatrics and Child Health, Aga Khan University for several years. The presence of this system ensures that women can be tracked during pregnancy and after delivery to ascertain their outcomes, which is critical to this project. This gestational age is most feasible to perform fetal Doppler that may help predict adverse outcomes. The eligibility criteria of the study are as follows:

Inclusion criteria:

• Pregnant woman coming to the ultrasound clinic between 22–34 weeks of gestation.
• Written informed consent obtained
• Resident of the study area

Exclusion criteria:

• Multiple gestation
• Known congenital anomaly in the fetus or newborn
• Refusal for the ultrasound
• Poor echocardiographic images for Doppler acquisition

Outcomes –

The primary outcomes of this study include:

• Stillbirth (baby born with no signs of life at or after 28 weeks of gestation) and
• Early neonatal mortality (death of a baby within the first 7 days of life).

The secondary outcomes include:

• IUGR – Fetal weight that is below the 10 ^th^ centile for gestational age on ultrasound
• Prematurity – Birth of a fetus < 37 weeks of gestation
• LBW – Birth weight < 2.5 kg
• Birth asphyxia – Delayed cry after at least 1 minute of life
• Early neonatal sepsis – Any 1 of the following signs: fever > 38 or < 35 Celsius, convulsions, lethargy, poor feeding, chest in drawing and tachypnea (respiratory rate > 60 breaths per minute) within 7 days of life ^9^

Sample size estimation – The sample size is based on outcomes of the pregnancies and not based on etiology of outcome. For the training of the machine learning, as much data as possible is required and this would be typically in the order of magnitude of thousands of patients. Given that this is not feasible in the pilot study, the training data will be mainly synthetically generated through a computational model of the fetal circulation. This allows the generation of thousands of 'individuals'. A recent randomized clinical trial conducted on machine learning in heart failure with 1106 patients with 20% having an adverse primary outcome and 5 different phenotypes, it was seen that above a sample size of 500 patients, the learned information becomes stable and doesn't change much ^10^. Additionally, we will be using socio-demographic, and pregnancy and labor characteristics, along with the Doppler, where the combined analysis should make the detection additionally more robust.

In the study area there are 30 stillbirths per 1000 total births while there are 30 neonatal deaths per 1000 live births. If 500 pregnant women are to be enrolled in the study duration, we expect to find approximately 25–30 adverse perinatal outcomes. To account for loss to follow up, we will inflate the sample size by 5% and enroll 525 pregnant women. Sample size was estimated from experience with the proposed machine learning approach, where stability of the learning phase was reached around 500 included patients ^18^, combined with the fact that data augmentation of the training set will be performed using the previously validated computational model of the fetal circulation ^7^.

Recruitment – Household surveillance will be done by community health workers (CHWs) in the catchment area in a sequential manner to identify pregnant women. Eligible women between 22–34 weeks of gestation on last menstrual period (LMP) will then have the purpose of the study explained to them and be invited for an ultrasound scan. At the clinic, all inclusion/exclusion criteria will be assessed. All screening procedures will be documented in the appropriate study forms, including logs and case report forms. Clinical assessments and findings will also be documented as appropriate. No identifying information will be retained for women who do not enroll in the study.

First and enrollment visit – The first visit window for the ultrasound scan will be between 22–26 weeks of gestation. This window is selected because pregnancies at risk may be better identified and also visualization of fetal cardiac flow is possible. Based on the rigorous surveillance, we anticipate that most women will be in this window. The following information will be collected:

• Socio-demographic information such as women’s age, educational status, occupation
• Medical history, including exposure to tobacco, gravidity, parity, past obstetric history (including number of miscarriages, stillbirths or neonatal deaths) and comorbid conditions (pre-eclampsia, diabetes mellitus)
• Maternal anthropometry (height, weight and mid-upper arm circumference)
• Maternal blood pressure
• Haemoglobin of the mother (using Hemocue) (Hemocue Pholometer Model 301; Ängelholm, Sweden)
• Symphysiofundal height will be measured
• Four chamber view of the mother’s heart
• Standard obstetric Doppler evaluation including the four primary ultrasound measures of fetal growth—head circumference, biparietal diameter, abdominal circumference, and femur length is routinely performed for all pregnant women presenting at the center. These parameters will be used to estimate fetal weight (automatically calculated by the machine).
• Focused Fetal Doppler Echocardiography using Vivid iq Premium machine (GE Healthcare, Chicago IL, USA) and a curvilinear probe – These would include transabdominal measurements of the Doppler velocity of the umbilical artery obtained in the free loop of the umbilical cord, the middle cerebral artery (MCA) will be obtained in the proximal third of the vessel ^19^, close to its origin in the internal carotid artery in an axial section of the fetal brain. The ductus arteriosus and fetal aortic isthmus (AoI) flow velocities will be obtained either in a sagittal view of the fetal thorax with a clear visualization of the aortic arch or in a cross section of the fetal thorax at the level of the 3-vessel and trachea view, and measurements of left and right ventricular outflow will be imaged in an apical or basal 5-chamber view of the heart ^20^.

Second visit – The second visit window of ultrasound scan will be between 30–34 weeks of gestation. This will be applicable for all women who have had their first scan during 22–26 weeks of gestation as well as newly enrolled women during 30–34 weeks of gestational age. We have identified this window based on the antenatal care seeking in this setting, where the first point of contact of the pregnant woman with the health system is in the third trimester. The same procedures as described in the first visit will be done. However, at the second visit, cardiotocography (CTG) and umbilical artery continuous wave Doppler image through UmbiFlow device will also be obtained. All scans of a woman (whether 1 or 2) will be included in the model at the relevant gestational age time points. 

Follow up – We will obtain contact information (phone number) from all enrolled women. A trained research midwife will be responsible for making phone calls, as well as performing household visits for immediate and early identification of the pregnancy outcome. A hotline number and emergency transfer for delivery will be provided to the enrolled women. In case of live births, a team of CHWs will perform home visits to the baby at post-natal day 1 and 6. At the first visit, labor and delivery information including questions related to delayed cry, meconium passage and hospital course (if any) will be collected and the weight and length of the baby will be recorded. In subsequent visits, the baby will be examined by CHW for questions related to early neonatal sepsis on standardized questionnaires. Facilitated referral will be provided in case the mother or newborn requires treatment. In case of a stillbirth, the same labor and delivery information will be collected at day of outcome.

**Figure 1.**
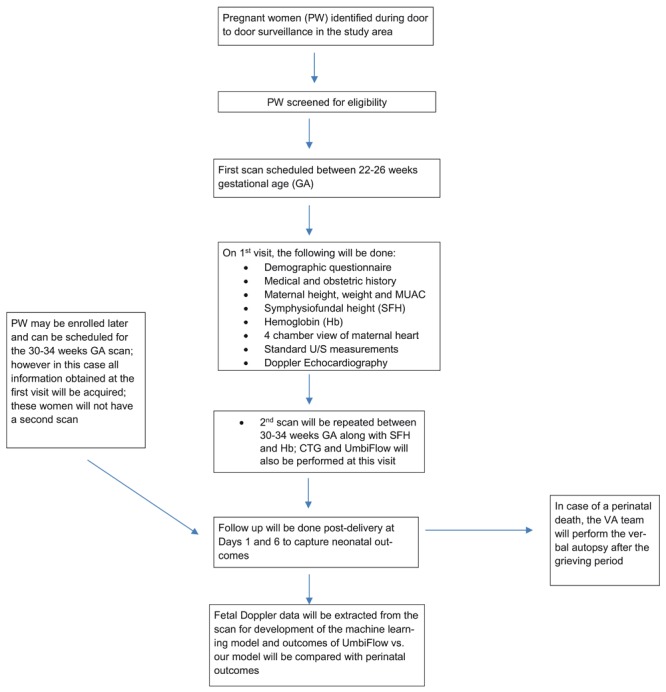
Study flow diagram.

Please refer to Figure 1 for the study flow diagram.

Verbal autopsy – The follow up team will inform the verbal autopsy (VA) team regarding any perinatal/neonatal death among the enrolled participants. The VA team will visit the family after the grieving period (at least 14 days after the death). VA will be performed on a structured questionnaire.

Withdrawal and early termination – The participant may voluntarily withdraw from the study for any reason at any time. The investigator may also withdraw mother or baby from the study in order to protect their safety if, in their opinion, continuing participation would jeopardize the participants health. Any participant withdrawal or early termination will be documented in the appropriate study forms

Training and quality control – The physician sonographer has voluntarily started her training under the supervision of a trained fetal echocardiographer at AKU for the past 6–8 weeks. This training will continue for another 6 weeks followed by one week observation of her skills at the PHC to perform fetal Dopplers. During the project duration, 10–15% of her images will be monitored every week (by 2 trained fetal echocardiographers) to ensure the quality and correct acquisition of the required images. Retraining will be offered if required.

**Figure 2.**
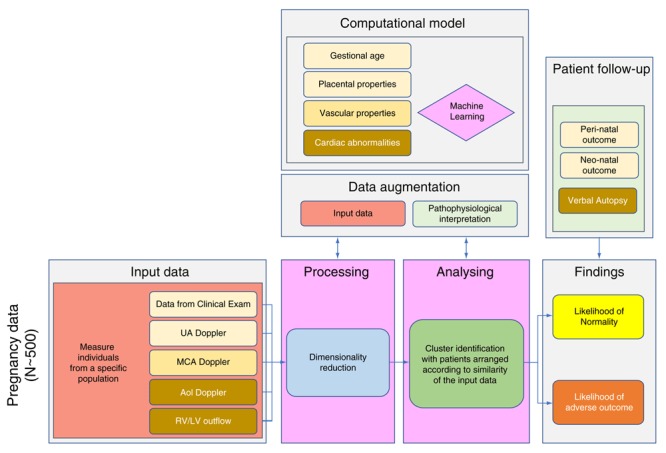
Machine learning algorithm for this study.

Data transfer and analysis – Fetal echo and ultrasound data will be interpreted jointly by the research team in a joint analysis meeting at AKU and Universitat Pompeu Fabra (UPF). Analysis will include developing the machine learning algorithm required to predict adverse perinatal outcomes. To fuse and order multivariate heterogeneous data, and be agnostic of any outcome, the Multiple Kernel Learning (MKL) methodology will be utilized in a non-supervised setting. The data would undergo non-linear dimensionality reduction to extract the most discriminate features from the multiple and rich input variables allowing for independent complex interrelation with abnormalities. Identifying clusters of individuals, after identifying the most relevant discriminate patterns in the population, and relating this to clinical findings and outcome, subsequently allows for the identification of phenogroups with similar outcome. When data from a new individual, not used for the learning phase, is available, the most similar patients from the learning phase can be identified (by projection of input data into the learned low-dimensional space) and the pheno-group membership can be quantified from which predicted outcome can be derived assuming it will be similar to the expected outcome of the group (refer to Figure 2).

Statistical analysis – Classical data analysis will be performed using SPSS version 20.0. Data will be reported as percentages and means +/- standard deviation where appropriate. Pearson chi-square will be used to compare qualitative data between groups. A student's t-test or Mann Whitney U test (based on assumption of normality) will be used to compare data between groups with presence or absence of adverse perinatal outcomes. Associations between various Doppler and model-based parameters and adverse perinatal outcome will be analyzed by multiple logistic regression. Machine learning will be performed using the Multiple Kernel Learning approach developed in-house and described previously ^18^.

Ethical concerns – This study has been approved by AKU Ethics Review Committee (4872-Ped-ERC-17). Written informed consent will be obtained from each pregnant woman to ensure that the she is informed of and fully understands what will and may happen to her and her baby while participating in the research study. If the woman requests for additional time or indicates the need for consultation with other family members, she will be provided time, as long as she remains in the studies window period. Informed consent will be obtained by the research assistant at the center before any kind of study data is obtained. Fetal ultrasonography will require additional 15–20 minutes which may cause the woman some inconvenience. This will be clearly explained prior to recruitment. The patient will also hold the right of requesting discontinuation of scan. For any abnormal finding on fetal ultrasound or pregnancy related complication, the woman will be examined by the PHC physician. If there is need for hospital referral, facilitated referral will be done to the pregnant woman’s hospital of choice, which usually is Jinnah Postgraduate Medical Center which is a public tertiary level health center in Karachi. There will be no additional cost to the family for participation in this project.

The results of this study will be shared through newspaper articles in the local media, continuous medical education seminars, conference proceedings and publications in well reputed journals.

## Discussion

**Figure 3.**
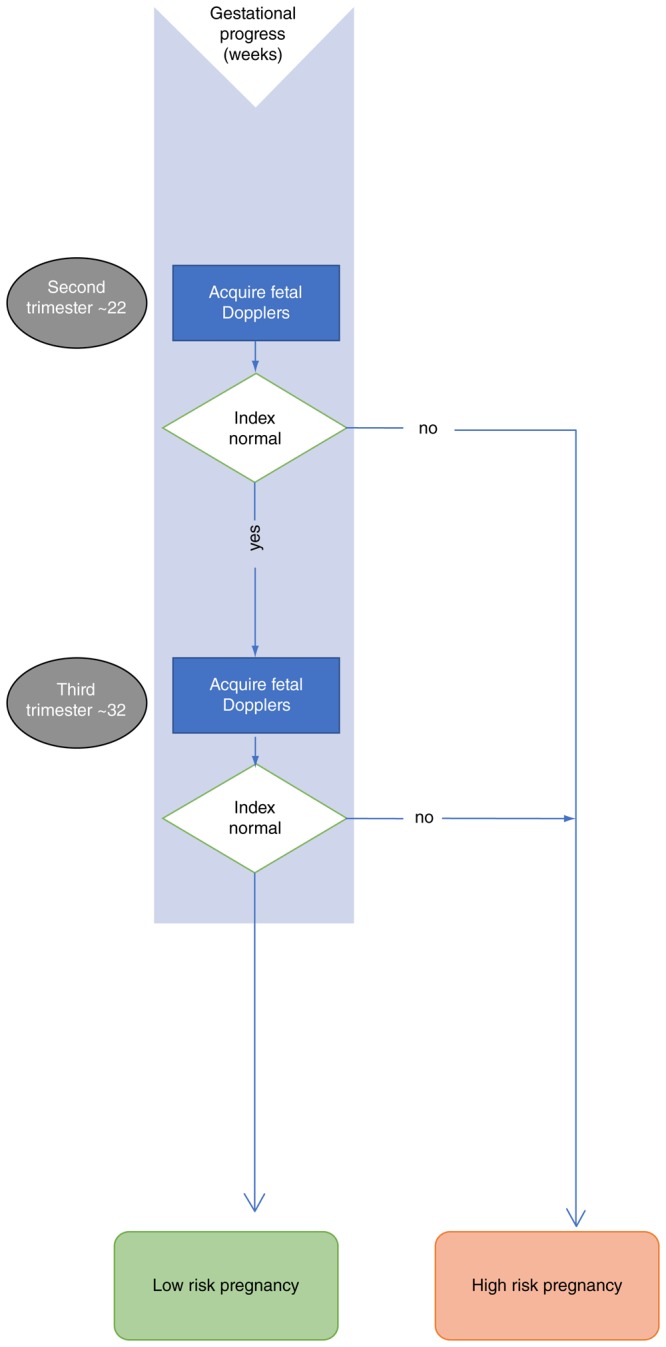
Proposed approach in the flow chart of clinical decision making.

The study will start recruitment in March 2018. Training the machine learning algorithms with data generated from the computational model and validating it in a population at risk would enable to identify the fetus and placenta that maybe compromised and be at risk for higher perinatal morbidity and mortality. This data would allow screening of pregnant women to detect at- risk and high-risk pregnancies. Early detection of these pregnancies would allow for timely referral to improve outcomes. Please refer to Figure 3 for the clinical decision making algorithm that may be proposed.
