# Supplementary material for: Machine learning from fetal flow waveforms to predict adverse perinatal outcomes: a study protocol
Source: Gates Open Res. 2018 Feb 12;2:8. [Version 1] doi: 10.12688/gatesopenres.12796.1 (PMC5974597; doi:10.12688/gatesopenres.12796.1)
Supplement: Peer review history

# Peer review: Referee response for version 1

**DOI:** 10.21956/gatesopenres.13857.r26427

**Published:** 2018-05-16

**Authors:** Shiraz A. Maskatia

**Affiliations:** 1 Department of Pediatrics, Stanford University School of Medicine, Stanford, CA, USA

The authors describe a thoughtful and detailed research plan which has the potential to significantly advance the field of maternal fetal medicine. They aim to use traditional fetal echocardiographic spectral Doppler acquisitions to develop a machine learning approach to identification of fetuses at high risk for perinatal demise. They are leveraging the expertise of fetal cardiologists and experts in machine-learning to develop this novel tool. The patients come from a high-risk population which increases the likelihood that clinical endpoints will be met. There is high likelihood that through participation in the study some mothers will have improved clinical outcomes, and there is very little likelihood for harm.

**General Comments:**

- I have no major concerns about this work.
- One general comment is that the many of fetuses will likely have 2 visits. However, many will only have one visit. It is not explained how this will be taken into account in the creation of the computational model to simulate patient visits or in the machine learning algorithm. I am confident that this will be adequately addressed based upon the qualifications of the investigators. Some explanation as to whether this poses a problem in the above analyses should be offered.
- I also wonder about how often this patient population delivers at home, at a delivery center or at a tertiary care institution. The difference in reporting of outcomes based upon these different settings may have to also be accounted for. While I am confident that the investigators will be diligent in following up these pregnancies that deliver at home, there may be some recall bias in those pregnancies that deliver at home, particularly in those that suffer neonatal mortality.

 

**Specific comments**

 Introduction:

- In the first paragraph, the high perinatal mortality rate is well described. I would recommend highlighting how the current methods to identify high-risk pregnancies fail to identify all of the pregnancies that result in perinatal mortality.   
- In the second paragraph, flow reversal in the aortic isthmus is mentioned as a finding associated with failure to “shift their right ventricular output from the pulmonary to the systemic circulation,” indicating cerebral hypoxia. I would explain this as the reverse. Due to fetal cerebral hypoxia, there is cerebrovascular dilation, resulting in steal of blood from the RV to the brain. This is due to an adaptive response of the brain to preserve oxygen delivery.
- In the fifth paragraph, I recommend changing the first sentence to read: “We hypothesize that the computational model of fetal hemodynamics will predict perinatal outcome independent of gestational age. An assessment which is independent of gestational age will be of high utility, as it will not rely on anthropometric measures, which can be challenging”
- Of note, I am wonder whether this model will prove to be independent of gestational age. It would make intuitive sense that fetuses who show derangements in fetal hemodynamics earlier in gestation will be higher risk.
- Page 2, last paragraph of introduction. I recommend changing the first sentence to: “The aim of this study is to obtain proof of concept for using a computational model of fetal hemodynamics, combined with machine learning based on Doppler patterns of the fetal cardiovascular, cerebral and placental flows, to identify fetuses at increased risk for adverse perinatal outcomes such as stillbirth, perinatal mortality, and other neonatal morbidities.”

Study protocol

 Inclusion criteria:

- Will mothers with chronic infections be included? These may also affect spectral Doppler patterns and perinatal outcome.

 

 Outcomes:

- Why is stillbirth defined as 28 weeks? The CDC definition starts at 20 weeks gestation.

 

 Secondary outcomes:

- Why is LBW used as an outcome measure as opposed to SGA (birthweight less than 10% for gestational age)?

 

 Sample size estimation:

- This is not my area of expertise. However, it seems to me that using a computational model to generate data defeats the purpose of generating training data. Based upon the authors credentials and expertise, I trust that this is sound methodology.

 

 Recruitment:

- No issues

 

 Focused Fetal Doppler Echocardiography:

- Why are the outflow tracts being measured? This was not previously mentioned as a measure of interest.

 

 Second visit:

- This is the first time that it is mentioned that serial measurements will be obtained. How will this be handled in the computational model and in the machine learning algorithm? Are there specific measures that need to be taken for each of these analyses that take into account repeated measures? Why is UmbiFlow only done at this later time point?

 

 Follow up:

- It may be useful to provide estimations on how often home delivery is performed in this community vs delivery at a birthing center, vs delivery at a tertiary care hospital.

I have read this submission. I believe that I have an appropriate level of expertise to confirm that it is of an acceptable scientific standard.

# Peer review: Referee response for version 1

**DOI:** 10.21956/gatesopenres.13857.r26252

**Published:** 2018-03-21

**Authors:** Sadia Malik

**Affiliations:** 1 Department of Pediatrics, University of Texas Southwestern, Dallas, TX, USA

This is a well written manuscript describing the planning stages of a prospective cohort study which will be conducted in Ibrahim Hyderi, a peri-urban settlement in south east of Karachi.

 The aim of this study is to provide proof of concept for using a computational model of fetal haemodynamics, combined with machine learning. This model will be based on Doppler patterns of the fetal cardiovascular, cerebral and placental flows with the goal to identify those fetuses at increased risk of adverse perinatal outcomes such as stillbirth, perinatal mortality and other neonatal morbidities.

 The eligibility criteria include pregnant women between 22-34 weeks who reside in the study area. Once enrolled, in addition to the performing fetal ultrasound to obtain Dopplers, data on socio-demographic, maternal anthropometry, haemoglobin and cardiotocography will be obtained on the pregnant women.

I have read this submission. I believe that I have an appropriate level of expertise to confirm that it is of an acceptable scientific standard.
